# The Effects of 4′-Esterified Resveratrol Derivatives on Calcium Dynamics in Breast Cancer Cells

**DOI:** 10.3390/molecules22111968

**Published:** 2017-11-14

**Authors:** Joshua A. Peterson, Hayden P. Doughty, Austin J. Eells, Trent A. Johnson, Jordan P. Hastings, Colton M. Crowther, Merritt B. Andrus, Jason D. Kenealey

**Affiliations:** 1 Department of Nutrition, Dietetics, and Food Science, Brigham Young University, Provo, UT 84602, USA; josh.peterson.587@gmail.com (J.A.P.); haydendoughty1@gmail.com (H.P.D.); austinjeells@gmail.com (A.J.E.); trentandjohnson@gmail.com (T.A.J.); jordanphastings@gmail.com (J.P.H.); 2 Department of Chemistry and Biochemistry, Brigham Young University, Provo, UT 84602, USA; coltoncrowther33@gmail.com (C.M.C.); mbandrus@chem.byu.edu (M.B.A.)

**Keywords:** resveratrol derivatives, calcium signaling, p53, cell viability, MDA-MB-231

## Abstract

Triple-negative breast cancer is a highly aggressive subtype of breast cancer. Frequently, breast cancer cells modulate their calcium signaling pathways to optimize growth. Unique calcium pathways in breast cancer cells could serve as a way to target tumorigenic cells without affecting normal tissue. Resveratrol has previously been shown to activate calcium signaling pathways. We use cell viability, single-cell calcium microscopy, and RT-PCR assays to determine the activity and mechanism of three different 4′-esterified resveratrol derivatives. We demonstrate that two of the derivatives reduce cell viability more effectively than resveratrol in MDA-MB-231 human breast cancer cells. The derivatives also activate similar pro-apoptotic calcium signaling pathways. In particular, the pivalated and butyrated resveratrol derivatives are intriguing putative chemotherapeutics because they are more effective at decreasing cell viability in vitro and inhibiting the plasma membrane Ca^2+^-ATPase, a protein that is often modulated in breast cancer.

## 1. Introduction

Breast cancer is the most common form of cancer in American women; in fact, one in eight women will be diagnosed with breast cancer during her lifetime [1]. Breast cancer is characterized, in part, by the presence or absence of three molecular markers: estrogen receptors, progesterone receptors, and HER2 [2]. Tumors that do not express any of these three markers are known as triple-negative breast cancer (TNBC) and are the most aggressive subtype [3]. In addition to its aggressiveness, TNBC does not respond well to standard chemotherapy, often becoming resistant to treatment. Therefore, it is important to identify novel chemotherapeutics with unique mechanisms to treat patients diagnosed with TNBC.

TNBCs have several unique modifications to calcium signaling pathways [4]. Calcium signaling occurs when the 100 nM calcium concentration of the cytosol ([Ca^2+^]*_i_*) is increased by an influx of calcium from the high calcium environments of the extracellular matrix (~1 mM) or the endoplasmic reticulum (ER) (~1 mM). Additionally, the [Ca^2+^]*_i_* can be increased by decreasing calcium efflux from the cytosol [5]. Calcium signaling is important for coordinating normal cellular activities such as mitosis, apoptosis, migration, cell metabolism, and immune function [6]. Cells utilize calcium signaling to regulate such a wide range of activities by varying the subcellular location and duration of the change in intracellular calcium concentration. Also, the calcium-binding proteins present during calcium signaling events can change the cellular interpretation of the calcium signal [7,8]. Since many of the cellular processes regulated by calcium signaling are modified in tumor cells, it is not surprising that the associated calcium signaling pathways are often changed in tumor cells [9]. These tumor-specific changes in calcium signaling pathways could be exploited to develop a non-toxic chemotherapeutic for TNBC.

*Trans*-resveratrol (RES) is a phytoalexin found in grapes, peanuts, Japanese knotweed, and many types of berries [10]. Clinical interest in RES began when it was identified as a cardioprotective component in red wine [11]. Subsequently, RES was discovered to be a putative, non-toxic [12] therapeutic for the treatment of diabetes, cardiovascular disease, and cancer [10,13,14]. RES showed enough promise as a therapeutic that several clinical trials were funded and completed. These clinical trials returned with mixed results due to the low bioavailability of RES [15].

**Figure 1:**
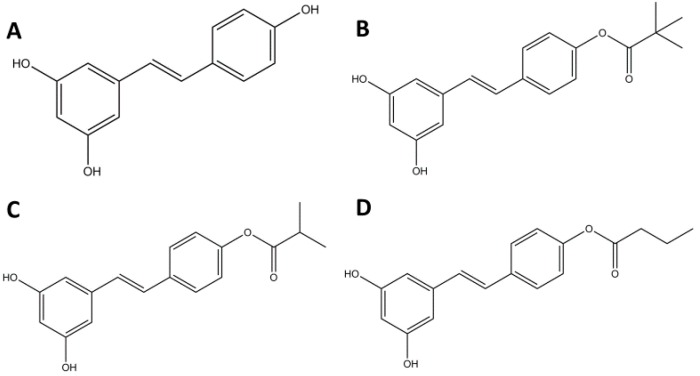
Structure of (**A**) *trans*-resveratrol, (**B**) 4′-Pivalate *trans*-resveratrol, (**C**) 4′-Isobutyrate *trans*-resveratrol, and (**D**) 4′-Butyrate *trans*-resveratrol.

Specifically, RES has been shown to activate apoptosis in tumor cells via increases in [Ca^2+^]*_i_* [16]. However, because RES has limited efficacy in in vivo studies and its pathways are largely unknown, our objective is to determine whether RES derivatives can be used to enhance the chemotherapeutic effects of RES, and to further elucidate the downstream effects of RES. Previous studies have demonstrated that RES esterified at the 4′-hydroxyl increases chemotherapeutic activity [17]. Therefore, in this study we esterified the 4′-hydroxy group with pivalate, butyrate, and isobutyrate groups (Figure 1) [18]. We tested the effects of the RES derivatives on tumor cell viability and on calcium signaling activity. We also investigated the upregulation of pro-apoptotic markers in response to RES. We found that certain RES derivatives induce significantly larger reductions in cell viability and elicit a more pronounced effect on several calcium signaling pathways in comparison to RES. We also found that RES upregulates two separate pro-apoptotic pathways through [Ca^2+^]*_i_* signaling.

## 2. Results

### 2.1. RES Derivatives Decrease Cell Viability

**Figure 2:**
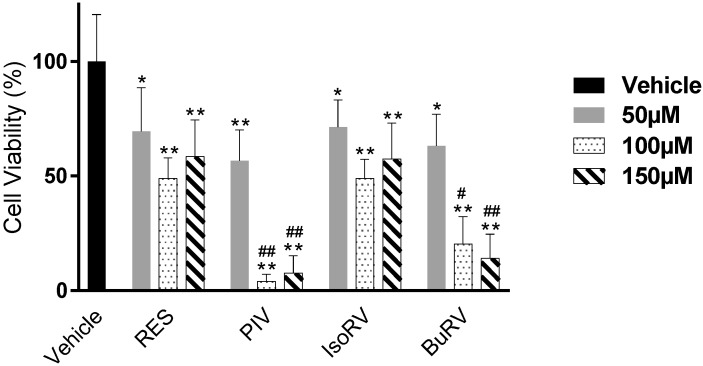
Resveratrol (RES) derivatives induce a decrease in breast cancer cell viability. MDA-MB-231 cells were treated with vehicle only (black), 50 μM (gray), 100 μM (dotted), or 150 μM (striped) of resveratrol (RES), 4′-pivalate resveratrol (PIV), 4′-isobutyrate resveratrol (IsoRV), or 4′-butyrate resveratrol (BuRV) for 48 h, and subsequently measured for viability with MTT. The data are representative of three biologic replicates conducted in triplicate. * indicates *p* < 0.05 compared to the vehicle control, ** indicates *p* < 0.01 compared to the vehicle control, ^#^ indicates *p* < 0.05 compared to RES at the equivalent concentration, and ^##^ indicates *p* < 0.01 compared to RES at the equivalent concentration.

Our interest in RES derivatives as potential chemotherapeutics and their pathways stems from the ability of RES to decrease cell viability in various cancer cell lines [10,19,20]. We utilized an MTT assay to determine the effects of RES derivatives on cell viability (Figure 2) in MDA-MB-231 cells after a 48-h treatment with concentrations of 50 μM, 100 μM, and 150 μM resveratrol, 4′-butyrate resveratrol (BuRV), 4′-isobutyrate resveratrol (IsoRV), or 4′-pivalate resveratrol (PIV). Compared to the vehicle control, we found that RES, BuRV, IsoRV, and PIV significantly decreased cell viability in MDA-MB-231. We also demonstrated differences between the effects of RES and RES derivatives on cell viability. We found that BuRV and PIV reduced cell viability significantly more than RES in MDA-MB-231 cells (14.14% and 7.70% cell viability after 150 μM BuRV and PIV treatment, respectively, compared to 58.45% in RES at the same concentration, all relative to a vehicle-treated control).

### 2.2. BAPTA-AM Mitigates RES-Induced Decreases in Cell Viability

**Figure 3:**
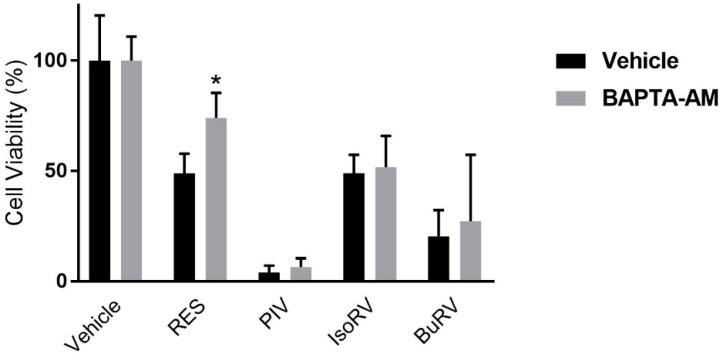
RES-induced decrease in cell viability is dependent on an increase in intracellular calcium. MDA-MB-231 cells were pretreated with 10 μM BAPTA-AM (gray bars) or a vehicle control (black bars). After the 15-min pretreatment, the cells were given the compound indicated on the *x*-axis. The data are representative of three biologic replicates conducted in triplicate. * indicates *p* < 0.05 compared to cells treated with the same RES or RES derivative treatment, but not pretreated with BAPTA-AM.

Based on previous research, we hypothesized that RES-mediated decreases in cell viability are dependent on Ca^2+^ signaling [16,21]. In order to test this hypothesis, we measured cell viability in MDA-MB-231 cells after a 48-h treatment with 100 μM RES, BuRV, IsoRV, or PIV following a 15-min pretreatment with 10 μM BAPTA-AM (Figure 3), an intracellular calcium-chelating agent. The 10 μM BAPTA-AM concentration was the highest that could be tolerated without BAPTA-AM inducing significant decreases in cell viability in MDA-MB-231 cells in the 48-h assay (data not shown). After treatment with 5 μM thapsigargin (TG), a sarcoendoplasmic calcium ATPase (SERCA) inhibitor, cells pretreated with 10 μM BAPTA-AM markedly diminished the increase in [Ca^2+^]*_i_* normally seen after treatment with TG (as determined by live-cell microscopy, Figure S2). We found that in RES-treated cells, compared to the control, the presence of BAPTA-AM attenuated the RES-induced decrease in cell viability by 25.1% percent. This result suggests that RES reduces cell viability through Ca^2+^ signaling. Interestingly, we found that pretreatment with BAPTA-AM did not significantly affect cell viability values after treatment with PIV, IsoRV, and BuRV.

### 2.3. RES Derivatives Induce Increases in [Ca^2+^]_i_

**Figure 4:**
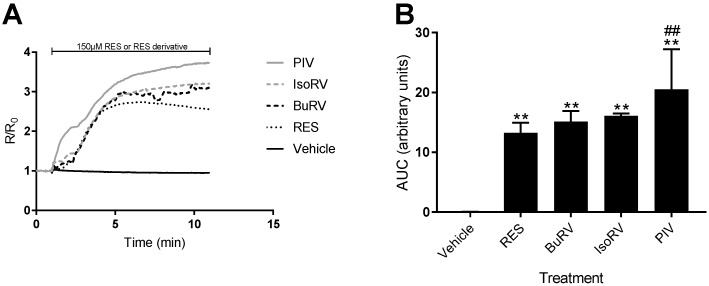
RES derivatives increase intracellular calcium levels. MDA-MB-231 cells were loaded with Fura-2, a calcium-sensitive fluorophore, to measure relative intracellular calcium levels. Fura-2-loaded MDA-MB-231 cells following 1 min of baseline collection were treated with 150 μM RES (dotted, black), BuRV (dash, black), IsoRV (dash, gray), PIV (solid, gray), and a vehicle control (solid, black). The data are represented as line traces (**A**) of the relative changes in intracellular calcium, and the area under the curve (AUC) quantified in the bar graph (**B**). Results are representative of three biologic samples. ** indicates *p* < 0.01 compared to the vehicle only control, and ^##^ indicates *p* < 0.01 compared to RES.

RES induces increases in cytosolic calcium concentration ([Ca^2+^]*_i_*) in several cancer cell lines [16,22]. Because we found that only the RES-induced decrease in cell viability was attenuated by inhibiting [Ca^2+^]*_i_*, we needed to determine whether the derivatives increased [Ca^2+^]*_i_* or acted by a different mechanism to decrease cell viability. Thus, we utilized live-cell microscopy to detect changes in relative [Ca^2+^]*_i_* after RES derivative addition. Treatment with 150 μM BuRV, IsoRV, and PIV induced increases in relative [Ca^2+^]*_i_* in MDA-MB-231 cells (Figure 4A). Changes in relative [Ca^2+^]*_i_* were quantified by finding the area under the curve (AUC) after treatment with RES derivatives in MDA-MB-231 cells (Figure 4B). In MDA-MB-231 cells (Figure 4B), we found that PIV induced an increase in relative [Ca^2+^]*_i_* that was significantly larger (by 21.66%) than the increase in relative [Ca^2+^]*_i_* induced by RES. In addition to analyzing the AUC for each compound, we determined the relative [Ca^2+^]*_i_* at the end of each assay. PIV also had an endpoint calcium concentration that was 46.03% (*p* < 0.05) higher than RES. This gives further evidence that PIV has a more pronounced effect on [Ca^2+^]*_i_* than RES.

### 2.4. RES Derivatives Inhibit Plasma Membrane Calcium ATPase (PMCA)

After demonstrating that RES derivatives induce increases in [Ca^2+^]*_i_*, we further explored the mechanisms by which RES derivatives may be increasing [Ca^2+^]*_i_*. [Ca^2+^]*_i_* increases can result from Ca^2+^ being retained in the cytosol due to impaired cytosolic Ca^2+^ exit, more Ca^2+^ entering the cytosol from internal or external Ca^2+^ stores, or a combination of these mechanisms. Previous studies have shown that RES activates a rise in [Ca^2+^]*_i_* by inhibition of the plasma membrane Ca^2+^-ATPase (PMCA) and/or by activation of G-proteins, presumably through RES binding to a member of the G-protein coupled receptor (GPCR) family [23,24].

To test the inhibitory activity of RES derivatives on PMCA, we utilized a protocol established by Samad et al. [25]. Briefly, cells were imaged in Ca^2+^-free media Hank’s buffered salt solution (HBSS) to restrict any changes in [Ca^2+^]*_i_* to Ca^2+^ coming from stores within the cell. We pretreated cells with 5 μM thapsigargin (TG), a sarcoendoplasmic reticular Ca^2+^-ATPase (SERCA) inhibitor (SERCA is responsible for pumping Ca^2+^ from the cytosol to the ER), 5 min prior to the addition of 150 μM RES, BuRV, IsoRV, or PIV. With SERCA inhibited by TG, any increase in [Ca^2+^]*_i_* after RES derivative treatment could be attributed to PMCA inhibition, as Ca^2+^ flows from the ER and accumulates in the cytosol with impaired exit to the extracellular space. In our assay, we calculated the AUC after treatment with RES derivatives to quantify the PMCA inhibition activity of each derivative.

**Figure 5:**
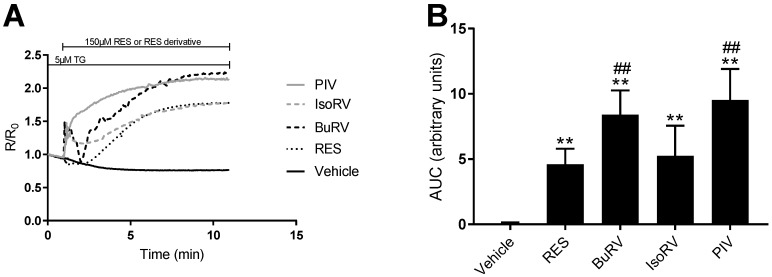
RES and RES derivatives inhibit plasma membrane calcium ATPase (PMCA). MDA-MB-231 cells were loaded with Fura-2, placed in a Ca^2+^-free media, and treated with the SERCA pump inhibitor, thapsigargin (TG), to deplete endoplasmic reticulum (ER) calcium. Placing the cells in Ca^2+^-free media and depleting ER calcium with TG isolates the activity of PMCA; thus, PMCA inhibition will be reflected by an increase in [Ca^2+^]*_i_*. Cells were then treated with 150 μM RES (dotted, black), PIV (solid, gray), IsoRV (dash, gray), BuRV (dash, black), or a vehicle (solid, black). The increase in [Ca^2+^]*_i_* is shown as a time trace (**A**) and quantified by the area under the curve (**B**). The data are a representation of three biologic replicates. ** indicates *p* < 0.01 compared to the vehicle control, and ## indicates *p* < 0.01 compared to RES.

Treatment with 150 μM BuRV, IsoRV, and PIV induced significant increases in relative [Ca^2+^]*_i_* following SERCA inhibition, indicating that these RES derivatives act as PMCA inhibitors in MDA-MB-231 cells (Figure 5). In MDA-MB-231 cells, BuRV and PIV induced increases in [Ca^2+^]*_i_* following SERCA inhibition that were significantly larger (by 82.60% and 107.05%, respectively) than increases in [Ca^2+^]*_i_* induced by RES after SERCA inhibition. These results suggest that, relative to RES, BuRV and PIV act as more potent PMCA inhibitors in MDA-MB-231 cells. The proportional decrease in cell viability induced by BuRV and PIV indicates that PMCA inhibition may play a role in the RES derivative anticancer effect.

### 2.5. The Effect of RES and Derivatives on ER Calcium Signaling

**Figure 6:**
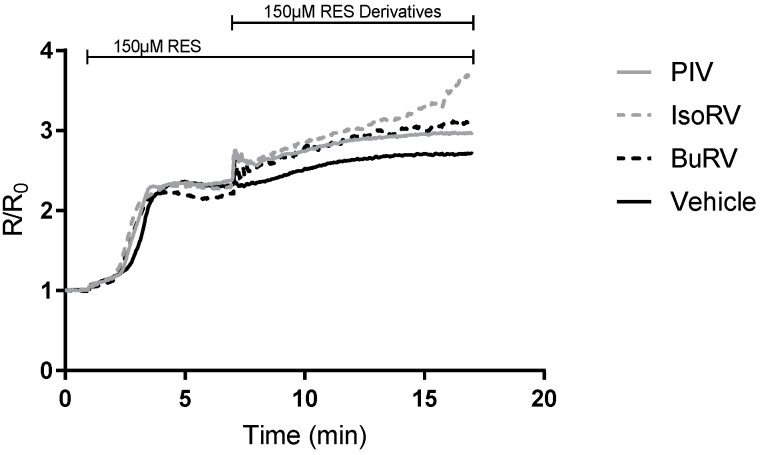
RES and RES derivatives activate the same calcium source(s) to increase [Ca^2+^]*_i_*. Fura-2-loaded MDA-MB-231 cells were treated with RES at 1 min and then with PIV (gray, solid), IsoRV (dash, gray), BuRV (dash, black), or a vehicle (black, solid) at 7 min. Each time trace is the average of three biologic replicates.

In addition to exploring the effects of RES and derivatives on PMCA, we identified the effects of RES derivatives on other aspects of calcium signaling pathways. To determine whether RES and the derivatives activate the same Ca^2+^ source(s) to increase [Ca^2+^]*_i_*, we measured changes in [Ca^2+^]*_i_* after treating MDA-MB-231 cells with RES, followed by RES derivatives 6 min later (Figure 6). The derivatives did not induce an additional rise in [Ca^2+^]*_i_* following RES treatment, indicating that RES and the derivatives mobilize the same calcium pool. RES has previously been shown to mobilize ER calcium; therefore, we explored the role of a pathway known to release calcium from the ER, namely, the phospholipase C (PLC) pathway. PLC cleaves phosphatidylinositol 4,5-bisphosphate (PIP_2_) to produce inositol triphosphate (IP_3_), which binds to the IP_3_ receptor (IP_3_R), a calcium channel that opens when IP_3_ binds to release ER Ca^2+^ [26].

**Figure 7:**
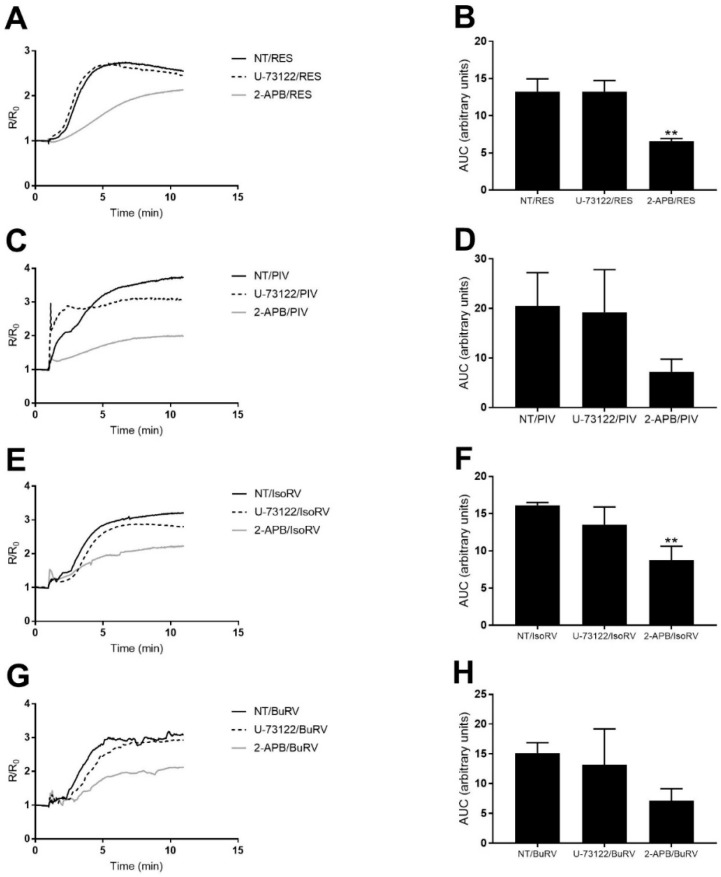
RES and RES derivatives activate calcium release from the IP_3_R independent of phospholipase C (PLC). To determine the source of calcium that leads to the increase in [Ca^2+^]*_i_*, MDA-MB-231 cells were pretreated with a PLC or an IP_3_R inhibitor, U-73122 (black dash) and 2-APB (solid gray), respectively. Cells were loaded with Fura-2 and pretreated with the indicated inhibitor for 10 min prior to treatment with RES (**A**,**B**); PIV (**C**,**D**); IsoRV (**E**,**F**); or BuRV (**G**,**H**). Cells pretreated with inhibitors and RES derivatives were compared to cells treated only with RES derivatives (solid black). Three biologic replicates are represented by a time trace (**A**,**C**,**E**,**G**) of average response during 10 min of data collection, and changes in [Ca^2+^]*_i_* are quantified by the area under the curve (**B**,**D**,**F**,**H**). ** indicates *p* < 0.01 compared to no pretreatment with the inhibitors.

We pretreated cells with 5 μM U-73122, a PLC inhibitor, 10 min prior to treatment with RES derivatives to determine the extent to which the increase in relative [Ca^2+^]*_i_* induced by RES derivatives is dependent on PLC. We compared the AUC after treatment with RES or derivatives to cells that were pretreated with U-73122 prior to treatment with RES or derivatives (Figure 7). In MDA-MB-231 cells, treatment with 150 μM RES, BuRV, IsoRV, and PIV after U-73122-mediated PLC inhibition did not induce changes in [Ca^2+^]*_i_* that were significantly different from changes in [Ca^2+^]*_i_* induced by treatment with RES, BuRV, IsoRV, and PIV alone. These results suggest that the increases in [Ca^2+^]*_i_* induced by RES derivatives are not dependent upon PLC, because inhibition of PLC by U-73122 does not significantly alter the changes in relative [Ca^2+^]*_i_* produced by RES derivatives.

We also tested the role of the IP_3_R in RES derivative-mediated increases in [Ca^2+^]*_i_* [26]. We pretreated cells with 100 μM 2-aminoethyl diphenylborinate (2-APB) (an IP_3_R antagonist) for 10 min prior to the addition of RES derivatives. We compared the AUC after treatment with RES or derivatives to cells that were pretreated with 2-APB prior to treatment with RES or derivatives (Figure 7). Treatment with 150 μM RES and IsoRV after IP_3_R inhibition induced increases in [Ca^2+^]*_i_* in MDA-MB-231 cells (Figure 7B,D,F,H) that were significantly smaller than increases in [Ca^2+^]*_i_* induced by RES, BuRV, IsoRV, and PIV without IP_3_R inhibition. Our results imply that the increases in relative [Ca^2+^]*_i_* induced by RES and IsoRV are dependent to some degree on IP_3_R-dependent Ca^2+^ release from the ER.

### 2.6. RES and Derivatives Decrease Cell Viability through an Additional, Non-p53-Dependent Pathway

**Figure 8:**
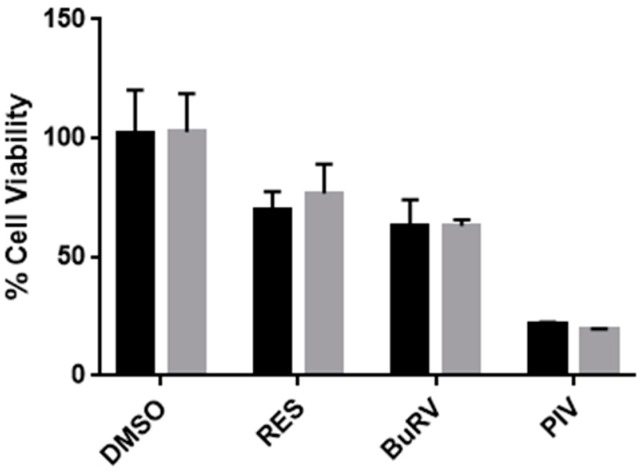
RES and derivatives decrease cell viability independent of p53. p53 in MDA-MB-231 cells was knocked down by p53-specific siRNA (gray) and non-targeting siRNA (black). Cells were then treated with vehicle, RES, BuRV, and PIV for 48 h and cell viability was measured. There was no statistical difference between the knockdown and control cells. This data is representative of two biologic replicates each performed in triplicate.

Our previous studies indicated that RES may decrease cell viability by increasing [Ca^2+^]*_i_* which, in turn, upregulates p53, a pro-apoptotic protein involved in tumor suppression [24]. To determine whether p53 is directly involved in the decrease in cell viability induced by RES derivatives, we performed an siRNA knockdown of p53 on MDA-MB-231 cells. The cells were treated for 48 h in a vehicle, 100 μM RES, BuRV, or PIV. We then measured cell viability in order to determine the dependence of RES, BuRV, and PIV on p53 to reduce cell viability (Figure 8). We found that RES, BuRV, and PIV all reduce cell viability to the same degree regardless of p53 knockdown. This data suggests that RES and RES derivatives decrease MDA-MB-231 cell viability independent of p53.

### 2.7. RES Induces Upregulation of NOXA, TP53INP, and RPM2B Gene Expression

**Figure 9:**
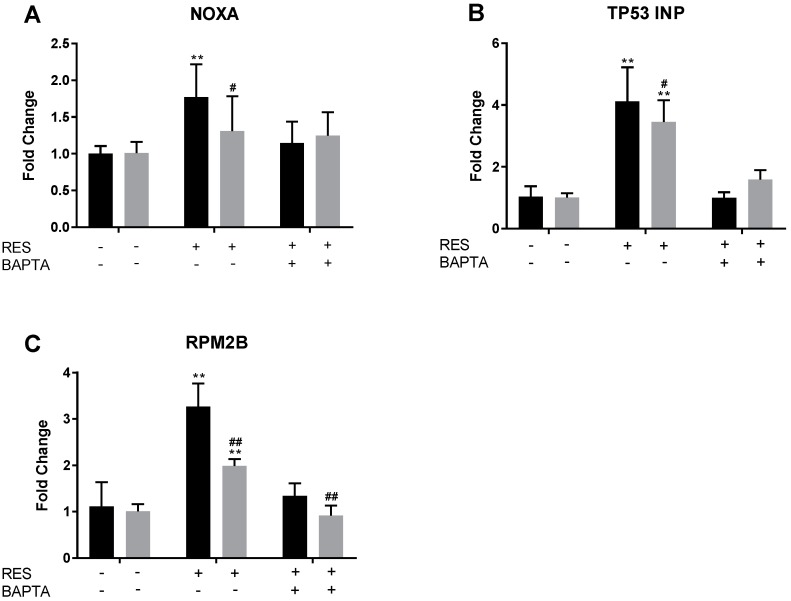
RES-induced upregulation of pro-apoptotic genes is dependent on an increase in [Ca^2+^]*_i_* and, to a lesser extent, p53. MDA-MB-231 cells were treated with p53-targeting siRNA (gray) and non-targeting siRNA (black) for 48 h. Subsequently, where indicated, cells were pretreated for 30 min with 20 μM BAPTA-AM (indicated by +), followed by treatment with 100 μM RES (indicated by +) for 6 h. RES induced a calcium-dependent increase in each of these three pro-apoptotic genes, NOXA (**A**), TP53 INP (**B**), and RPM2B (**C**). These data represent three biologic replicates that were each performed in triplicate. ** indicates *p* < 0.01 compared to the untreated control, ^#^ indicates *p* < 0.05 compared to the non-targeting siRNA control treated with the same treatment, and ^##^ indicates *p* < 0.01 compared to the non-targeting siRNA control treated with the same treatment.

To further elucidate the potential pathway of RES and its derivatives, we performed an siRNA knockdown of p53 on MDA-MB-231 cells. The cells were treated with siRNA for 48 h and then with BAPTA-AM for 30 min when indicated. Finally, cells were treated with 100 μM RES or a vehicle control. After 6 h, RT-PCR was performed on several pre-apoptotic markers, including NOXA, TP53INP, and RPM2B. Previous studies indicated that these p53-regulated genes were upregulated in response to RES treatment [24]. In this study, we determine the RES-induced Ca^2+^ dependence of these genes. Results indicate that regardless of p53 knockdown, TP53INP, RPM2B, and NOXA are upregulated in the presence of RES (Figure 9). The presence of BAPTA-AM mitigated the upregulation of these proteins in both the wild-type and knockdown MDA-MB-231 cells. This seems to be a further indication that RES (and possibly its derivatives) upregulates another pro-apoptotic pathway containing TP53INP, RPM2B, and NOXA through calcium signaling.

## 3. Discussion

In an effort to identify more efficacious, RES-based chemotherapeutics, we considered the effects of RES, BuRV, IsoRV, and PIV on cell viability, calcium signaling activity, p53, and pro-apoptotic markers in MDA-MB-231 breast cancer cells. Our data indicates that RES, BuRV, IsoRV, and PIV are effective at reducing cell viability, with BuRV and PIV reducing cell viability more than RES. While RES, BuRV, IsoRV, and PIV significantly reduce cell viability (as compared to the vehicle treatment), only RES-treated cells have cell viability restored when pretreated with BAPTA-AM. The ability of RES derivatives to induce changes in breast cancer cell viability, apparently independent of calcium signaling, may indicate that the 10 μM BAPTA-AM was insufficient to block the increase in [Ca^2+^]*_i_* induced by the RES derivatives. Alternately, the RES derivatives may utilize a calcium-independent pathway to reduce cell viability, unlike RES.

PIV induced total [Ca^2+^]*_i_* increases (as determined by the AUC) significantly larger than those induced by RES, which correlates with the increased effect of PIV on cell viability. Our PMCA inhibition assay revealed that RES as well as all RES derivatives under consideration inhibited PMCA in MDA-MB-231 cells, while only BuRV and PIV were found to be more potent inhibitors of PMCA than RES. Our IP_3_R and PLC inhibition assays also indicated that the RES and IsoRV-induced increases in [Ca^2+^]*_i_* show some level of dependency upon IP_3_R stimulation, but not PLC stimulation. Overall, our data indicates that, much like RES, ER-dependent Ca^2+^ release is utilized by RES derivatives to increase [Ca^2+^]*_i_*. Although decreases in cell viability induced by RES derivatives were not attenuated by BAPTA-AM treatment, RES derivatives activate an increase in [Ca^2+^]*_i_*. The experiments we conducted examining how Ca^2+^ accumulates in the cytosol provide further information on how RES and RES derivatives may affect [Ca^2+^]*_i_* and cell viability through the same pathway. However, it is unlikely that the molecular mechanism for RES and its derivatives is solely explained by calcium signaling. RES is a multifunctional small molecule that has been shown to activate several proapoptotic pathways [10], and it is likely that RES derivatives do so as well.

This study is limited because we only used a single breast cancer cell line. Further studies are needed to determine the robustness of the chemotherapeutic activity of these RES derivatives. Ultimately, the objective of studies with RES derivatives is to discover derivatives that retain tumor-specific RES activity, but are not limited by bioavailability like RES. PIV seems the best candidate from this study to move onto preclinical and clinical studies because of its increased efficacy.

*Tert*-butyl modifications of chemotherapeutics have been previously shown to pass the blood brain barrier more easily than their non-modified counterparts. Further, the *tert*-butyl, or pivalate, group has been shown to be resistant to hydrolysis [27]. In this study, we demonstrate that PIV, a *tert*-butyl-modified RES derivative, significantly decreases cell viability and modulates [Ca^2+^]*_i_* in MDA-MB-231 cell lines. TNBCs have uniquely modified calcium signaling pathways [28] and metastasize to the brain more frequently than other subtypes of breast cancer [29]. The properties of *tert*-butyl modifications to RES potentially make PIV a good candidate for the treatment of TNBC.

Our experiments identified RES derivatives that reduce cell viability more effectively than RES (BuRV and PIV) in MDA-MB-231. We demonstrated that BuRV, IsoRV, and PIV modulate the calcium signal, in some cases more so than RES. While lack of bioavailability stands as a major impediment to RES in clinical use, PIV and BuRV could potentially serve as chemotherapeutics because of their increased ability to decrease cell viability. Further study is required to uncover the cellular mechanisms that cause MDA-MB-231 cells to react differently to treatment with RES derivatives. Additional study is also needed to further elucidate the exact pro-apoptotic pathway of RES. As the viability-reducing mechanisms of BuRV and PIV are discovered, RES derivatives may be harnessed as potential non-toxic chemotherapeutics.

## 4. Materials and Methods

### 4.1. Materials

The MDA-MB-231 human breast cancer (HTB-26) cell line was purchased from ATCC (Manassas, VA, USA). The passage numbers of cells used in experiments ranged from 5 to 20. thapsigargin (10522) (TG), fura-2-acetoxymethyl ester (14591) (Fura-2), *N*,*N*′-[1,2-ethanediylbis[(oxy-2,1-phenylene)]]bis[*N*-[2-(acetyloxy)methoxyl]-2-oxoethyl]-bis[(acetyloxy)methyl] ester (BAPTA-AM-Acetoxymethyl ester) (15551) (BAPTA-AM), and *trans*-resveratrol (70675) (RES) were purchased from Cayman Chemical (Ann Arbor, MI, USA). U-73122 (J62898) and 2-Aminoethyl diphenylborinate (A16606) (2-APB) were purchased from Alfa Aesar (Ward Hill, MA, USA). 3-(4,5-Dimethylthiazol-2-yl)-2,5-diphenyltetrazolium bromide (AC158990050) (MTT) was purchased from Acros Organics (Morris Plains, NJ, USA). 4′-Butyrate resveratrol (BuRV), 4′-Isobutyrate resveratrol (IsoRV) and 4′-Pivalate resveratrol (PIV) were synthesized by the Andrus lab in the Department of Chemistry and Biochemistry at Brigham Young University [18].

### 4.2. Cell Culture

MDA-MB-231 cells were cultured in Dulbecco’s Modified Eagle medium (DMEM). DMEM was supplemented with 10% heat-inactivated fetal bovine serum (FBS) and 1% penicillin/streptomycin. MDA-MB-231 cells were cultured at 37 °C in 5% CO_2_.

### 4.3. Cell Viability

Cell viability was determined using an MTT assay. Cell cultures were plated at 10,000 cells per well in a Greiner Bio-One Cellstar 96-well plate (Greiner Bio-One, Monroe, NC, USA). Cells were grown for 24 h after initial seeding. After 24 h, cell culture media was removed and fresh media with treatments of individual RES derivatives at the indicated concentrations in 1.5% dimethyl sulfoxide (DMSO) were added. In experiments involving BAPTA-AM-treated cells, after the 24 h initial seeding time had passed, cell culture media was removed, and 10 μM BAPTA-AM in cell culture media was added. After 15 min, the BAPTA-AM solution was removed and fresh media with the indicated concentrations of RES derivatives was added. After 48 h, 20 μL of 5 mg/mL MTT was added to each well. Cells were then incubated for 3.5 h at 37 °C in 5% CO_2_. Media and MTT were removed, after which 150 μL of MTT solvent was added. Cells were agitated on an orbital shaker at 75 rpm for 15 min before absorbance was read at 590 nm with a reference filter at 620 nm on a BMG LABTECH FLUOstar OPTIMA plate reader (BMG LABTECH Inc., Cary, NC, USA). All experiments were performed in triplicate (*n* = 3).

### 4.4. Intracellular Calcium Imaging

Fura-2 loading and experimental protocols performed were similar to those performed by Peterson et al. [23]. Cells were prepared for imaging by plating to achieve a final cell density of 80,000 cells per well. Cells were plated in an 8-well chamber Lab-Tek #1.0 borosilicate coverglass (Thermo Scientific, Rochester, NY, USA). We recorded no significant changes due to variation in cell density at initial seeding or the amount of time between seeding and imaging. In preparation for imaging, media was removed and cells were washed twice with 1:1 PBS:FBS solution. Cells were then incubated in 8 μM Fura-2 in Ringer’s solution (NaCl 150 mM, glucose 10 mM, HEPES 5 mM, KCl 5 mM, MgCl_2_ 1 mM, CaCl_2_ 2 mM, pH 7.4). After 15 min (MDA-MB-231) of incubation at 37 °C in 5% CO_2_ in Fura-2 solution, the Fura-2 solution was removed and replaced with fresh Ringer’s solution. Fura-2-loaded cells were allowed to equilibrate in fresh Ringer’s solution for 30 min prior to imaging. Changes in [Ca^2+^]*_i_* were measured using Fura-2, a Ca^2+^-binding dual-excitation fluorophore that excites at 340 nm when bound to Ca^2+^ and at 380 nm when not bound to Ca^2+^ [30]. Fura-2 emits at 510 nm in both Ca^2+^-bound and Ca^2+^-unbound states. Fura-2-loaded cells were alternately excited at 340 nm and 380 nm while emission intensity was measured at 510 nm. By calculating the ratio of 340 nm/380 nm emission signal intensity, we were able to determine relative changes in [Ca^2+^]*_i_* following treatment with RES derivatives. For experiments studying plasma membrane Ca^2+^-ATPase (PMCA) inhibition, fresh Ringer’s solution was removed and replaced with a Ca^2+^-free buffer, Ca^2+^- and Mg^2+^-free Hank’s Balanced Salt Solution (HBSS).

Following Fura-2 loading, cells were imaged with an Olympus IX51 inverted microscope. Treatments were added manually in 100-μL volumes. All RES and RES derivatives were in 1.5% DMSO in Ringer’s solution, with the exception of experiments studying PMCA inhibition in which HBSS was used in place of Ringer’s solution as a vehicle. Pretreatments of 2-APB were also in 1.5% DMSO in Ringer’s solution, while TG and U-73122 pretreatments used Ringer’s solution alone as a vehicle. In all experiments, cells were imaged for 1 min prior to the addition of treatment to establish a baseline. In experiments studying PMCA inhibition, cells were incubated in inhibitor (TG) for 5 min prior to RES derivative addition. In experiments studying phospholipase C (PLC) and inositol triphosphate receptor (IP_3_R) inhibition, cells were incubated in inhibitors (U-73122 and 2-APB, respectively) for 10 min prior to the addition of RES derivatives.

Images acquired were analyzed using CellSens software (version number 1.11, Olympus, Tokyo, Japan from Olympus. Regions of interest (ROI) were identified representing one cell each. At least 10 ROIs per experiment were chosen and analyzed in order to measure changes in relative [Ca^2+^]*_i_*. The results of the analysis were normalized for each ROI and used to find the mean change in relative [Ca^2+^]*_i_* for each experiment. Each experiment was performed in triplicate (*n* ≥ 3). The calcium traces were quantified by determining the area under the curve (AUC) or total calcium response after treatment addition using GraphPad Prism 7 (La Jolla, CA, USA).

### 4.5. siRNA-Mediated Knockdown of p53 Gene Expression

The siRNA knockdown of p53 in MDA-MB-231 was performed as described previously [31]. Briefly, cells were allowed to adhere overnight in 6-well plates. DharmaFECT 4 (0.1 μL/well, Dharmacon, (Lafayette, CO, USA) was used to transfect cells with Dharmacon ON-TARGETplus^TM^ SMARTpool siRNA. Transfection media was prepared according to the manufacturer’s instructions and contained 8% FBS. The siRNAs used in this study were those for p53 (sip53, L-003329-00) and the non-targeting control siRNA (siNT, D-001810-10). All experiments had mRNA knockdown >70% at 6 h post-siRNA (Figure S1).

### 4.6. PCR

Cells were plated at a density of 500,000 per Petri dish and allowed to adhere to the dish for 48 h. Cells were pretreated with 20 μM BAPTA-AM or 1% DMSO control for 30 min, then treated with 100 μM RES or DMSO control for 6 h. RNA was then extracted using Qiagen RNeasy Plus Mini Kit according to the manufacturer’s protocol. Sample integrity was checked using an agarose RNA gel and nanodrop. RNA samples were used to create cDNA with an Applied Biosystems High Capacity Reverse Transcription Kit according to the manufacturer’s instructions. Concentrations of samples were obtained using nanodrop data. PCR was conducted using the primers shown in Table S1 and the iTaq Universal SYBR Green Supermix. Samples were run in Step One Plus (Applied Biosystems, Foster City, CA, USA).

### 4.7. Statistical Analysis

The statistical significance, where indicated, was determined by a two-way analysis of variance (ANOVA) with Bonferroni correction. Statistically significant differences between RES or a derivative treatment and the vehicle treatment are indicated by * if *p* < 0.05 and ** if *p* < 0.01. Statistically significant differences between RES derivative treatment and RES treatment are indicated by # if *p* < 0.05 and ## if *p* < 0.01. Additionally, in experiments with siRNA treatments, * indicates *p* < 0.05 and ** indicates *p* < 0.01 in comparison to the vehicle control. # indicates *p* < 0.05 and ## indicates *p* < 0.01 in comparison to the non-targeting control.

## Supplementary Materials

The supplementary materials are available online.

## References

[1] Siegel R.L., Miller K.D., Jemal A. (2016). Cancer statistics, 2016. CA Cancer J. Clin..

[2] Chavez K.J., Garimella S.V., Lipkowitz S. (2010). Triple negative breast cancer cell lines: One tool in the search for better treatment of triple negative breast cancer. Breast Dis..

[3] Foulkes W.D., Smith I.E., Reis-Filho J.S. (2010). Triple-Negative Breast Cancer. N. Engl. J. Med..

[4] Azimi I., Roberts-Thomson S.J., Monteith G.R. (2014). Calcium influx pathways in breast cancer: Opportunities for pharmacological intervention. Br. J. Pharmacol..

[5] Berridge M.J., Bootman M.D., Roderick H.L. (2003). Calcium signalling: Dynamics, homeostasis and remodelling. Nat. Rev. Mol. Cell Biol..

[6] Berridge M.J., Lipp P., Bootman M.D. (2000). The versatility and universality of calcium signalling. Nat. Rev. Mol. Cell Biol..

[7] Berridge M.J. (1997). The AM and FM of calcium signalling. Nature.

[8] Bootman M.D., Collins T.J., Peppiatt C.M., Prothero L.S., MacKenzie L., De Smet P., Travers M., Tovey S.C., Seo J.T., Berridge M.J. (2001). Calcium signalling—An overview. Seminars in Cell & Developmental Biology.

[9] Kadio B., Yaya S., Basak A., Dje K., Gomes J., Mesenge C. (2016). Calcium role in human carcinogenesis: A comprehensive analysis and critical review of literature. Cancer Metastasis Rev..

[10] Aggarwal B.B., Bhardwaj A., Aggarwal R.S., Seeram N.P., Shishodia S., Takada Y. (2004). Role of resveratrol in prevention and therapy of cancer: Preclinical and clinical studies. Anticancer Res..

[11] Renaud S.D., de Lorgeril M. (1992). Wine, alcohol, platelets, and the French paradox for coronary heart disease. Lancet.

[12] Cottart C.H., Nivet-Antoine V., Laguillier-Morizot C., Beaudeux J.L. (2010). Resveratrol bioavailability and toxicity in humans. Mol. Nutr. Food Res..

[13] Wu J.M., Hsieh T.Ä. (2011). Resveratrol: A cardioprotective substance. Ann. N. Y. Acad. Sci..

[14] Bhatt J.K., Thomas S., Nanjan M.J. (2012). Resveratrol supplementation improves glycemic control in type 2 diabetes mellitus. Nutr. Res..

[15] Tome-Carneiro J., Larrosa M., González-Sarrías A., Tomás-Barberán F.A., García-Conesa M.T., Espín J.C. (2013). Resveratrol and clinical trials: The crossroad from in vitro studies to human evidence. Curr. Pharm. Des..

[16] McCalley A.E., Kaja S., Payne A.J., Koulen P. (2014). Resveratrol and calcium signaling: Molecular mechanisms and clinical relevance. Molecules.

[17] Wong Y., Osmond G., Brewer K.I., Tyler D.S., Andrus M.B. (2010). Synthesis of 4′-ester analogs of resveratrol and their evaluation in malignant melanoma and pancreatic cell lines. Bioorg. Med. Chem. Lett..

[18] Acerson M.J., Fabick K.M., Wong Y., Blake C., Lephart E.D., Andrus M.B. (2013). A new synthesis of 4′-resveratrol esters and evaluation of the potential for anti-depressant activity. Bioorg. Med. Chem. Lett..

[19] Gwak H., Kim S., Dhanasekaran D.N., Song Y.S. (2016). Resveratrol triggers ER stress-mediated apoptosis by disrupting N-linked glycosylation of proteins in ovarian cancer cells. Cancer Lett..

[20] Li P., Yang S., Dou M., Chen Y., Zhang J., Zhao X. (2014). Synergic effects of artemisinin and resveratrol in cancer cells. J. Cancer Res. Clin. Oncol..

[21] Ma X., Tian X., Huang X., Yan F., Qiao D. (2007). Resveratrol-induced mitochondrial dysfunction and apoptosis are associated with Ca^2+^ and mCICR-mediated MPT activation in HepG2 cells. Mol. Cell. Biochem..

[22] Sareen D., Darjatmoko S.R., Albert D.M., Polans A.S. (2007). Mitochondria, calcium, and calpain are key mediators of resveratrol-induced apoptosis in breast cancer. Mol. Pharmacol..

[23] Peterson J.A., Oblad R.V., Mecham J.C., Kenealey J.D. (2016). Resveratrol inhibits plasma membrane Ca^2+^-ATPase inducing an increase in cytoplasmic calcium. Biochem. Biophys. Rep..

[24] Van Ginkel P.R., Yan M.B., Bhattacharya S., Polans A.S., Kenealey J.D. (2015). Natural products induce a G protein-mediated calcium pathway activating p53 in cancer cells. Toxicol. Appl. Pharmacol..

[25] Samad A., James A., Wong J., Mankad P., Whitehouse J., Patel W., Alves-Simoes M., Siriwardena A.K., Bruce J.I. (2014). Insulin protects pancreatic acinar cells from palmitoleic acid-induced cellular injury. J. Biol. Chem..

[26] Foskett J.K., White C., Cheung K.H., Mak D.O. (2007). Inositol trisphosphate receptor Ca^2+^ release channels. Physiol. Rev..

[27] Genka S., Deutsch J., Shetty U.H., Stahle P.L., John V., Lieberburg I.M., Ali-Osman F., Rapoport S.I., Greig N.H. (1993). Development of lipophilic anticancer agents for the treatment of brain tumors by the esterification of water-soluble chlorambucil. Clin. Exp. Metastasis.

[28] Peters A.A., Milevskiy M.J.G., Lee W.C., Curry M.C., Smart C.E., Sannus J.M., Reid L., da Silva L., Marcial D.L., Dray E. (2016). The calcium pump plasma membrane Ca^2+^-ATPase 2 (PMCA2) regulates breast cancer cell proliferation and sensitivity to doxorubicin. Sci. Rep..

[29] Dent R., Hanna W.M., Trudeau M., Rawlinson E., Sun P., Narod S.A. (2009). Pattern of metastatic spread in triple-negative breast cancer. Breast Cancer Res. Treat..

[30] Grynkiewicz G., Poenie M., Tsien R.Y. (1985). A new generation of Ca^2+^ indicators with greatly improved fluorescence properties. J. Biol. Chem..

[31] Curry M.C., Luk N.A., Kenny P.A., Roberts-Thomson S.J., Monteith G.R. (2012). Distinct regulation of cytoplasmic calcium signals and cell death pathways by different plasma membrane calcium ATPase isoforms in MDA-MB-231 breast cancer cells. J. Biol. Chem..

