# Supplementary material for: The Effects of 4′-Esterified Resveratrol Derivatives on Calcium Dynamics in Breast Cancer Cells
Source: Molecules. 2017 Nov 14;22(11):1968. doi: 10.3390/molecules22111968 (PMC6150182; doi:10.3390/molecules22111968)
Supplement: Supplementary file 1 [file molecules-22-01968-s001.pdf]

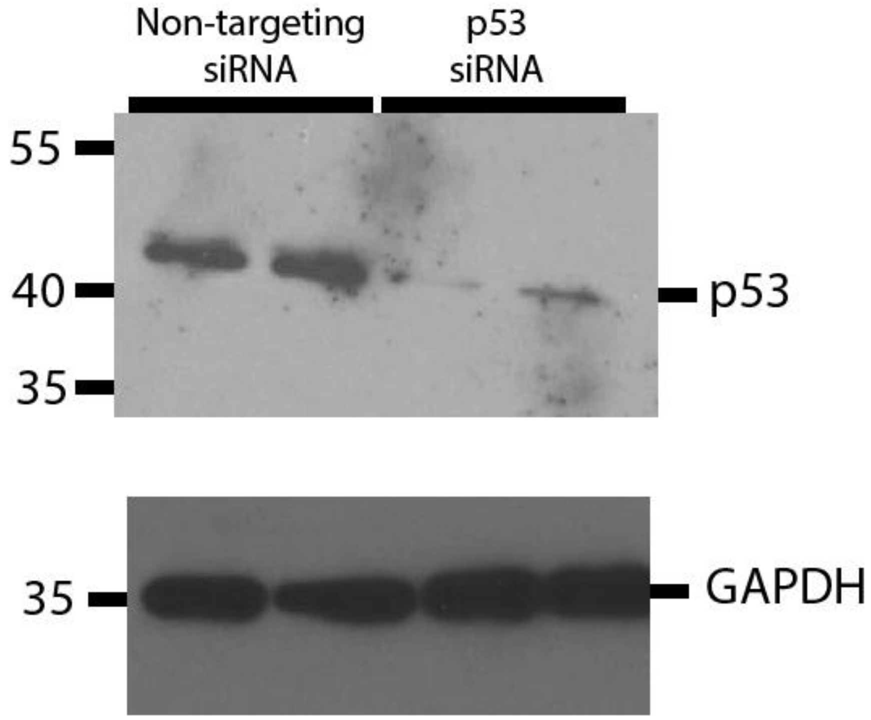

Figure S1. The knockdown of p53 with siRNA.

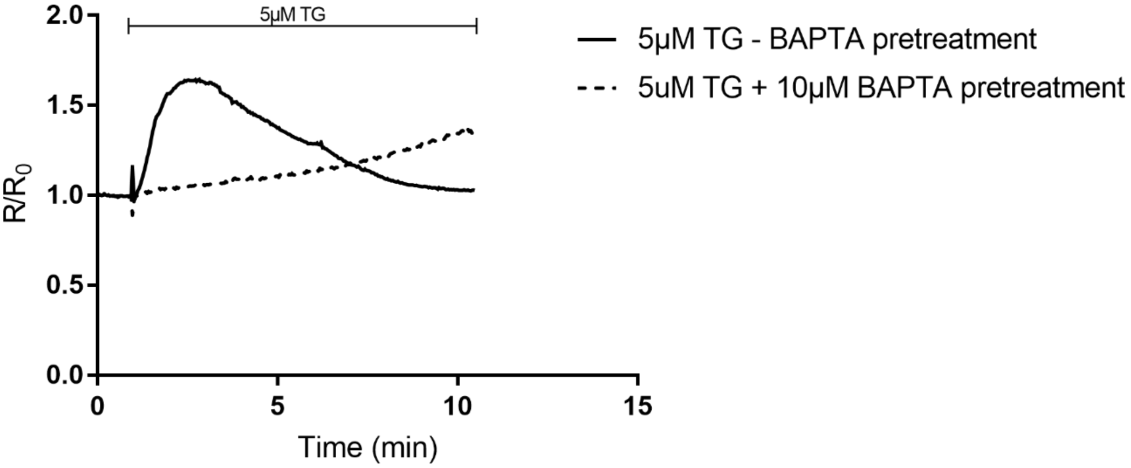

Figure S2. Inhibition of the calcium signal induced by thapsigargin with 10 uM BAPTA.

Table S1.

Gene	Primer sequence
TP53INP	Forward: TAGTCCCAGGGCCAGGAAAA<br>Reverse: TATCCACTGGGAAGGGCGAA
NOXA	Forward: GCTGGAAGTCGAGTGTGCTA<br>Reverse: GGAGTCCCCTCATGCAAGTT
RPM2B	Forward: GTAGCTTCGGCGGAGTCTG<br>Reverse: ACCGGCGAGAACTCTTTCTT
